# Predicting post-ESD bleeding in elderly patients with early gastrointestinal cancer: an explainable model based on endoscopic features

**DOI:** 10.3389/fmed.2026.1780097

**Published:** 2026-08-12

**Authors:** Rui Liu, Jianzhong Ma, Zhengwu Zhou, Yuanyuan Xie, Jiaojiao Su

**Affiliations:** 1 Department of Emergency Surgery, Lu'an Hospital of Anhui Medical University, Lu'an, China; 2 Department of Gastroenterology, Lu'an Hospital of Anhui Medical University, Lu'an, China

**Keywords:** bleeding, early gastrointestinal cancer, elderly, endoscopic submucosal dissection, machine learning, model interpretability

## Abstract

**Background:**

Endoscopic submucosal dissection (ESD) has emerged as the treatment of choice for early gastrointestinal cancers due to its minimally invasive nature, short recovery period, and brief hospitalization. However, postprocedural bleeding remains a frequent complication that may adversely affect clinical outcomes, particularly in elderly patients. Accurate prediction of bleeding risk after ESD is therefore essential to guide timely preventive interventions, mitigate complication rates, and ultimately enhance patient prognosis.

**Objective:**

To develop and interpret a machine learning model based on endoscopic features for predicting post–endoscopic submucosal dissection bleeding in elderly patients with early gastrointestinal cancer, using SHAP (SHapley Additive exPlanations) to provide explainable insights. This model is intended to support clinicians in early risk stratification and to facilitate individualized perioperative management aimed at improving outcomes.

**Methods:**

We conducted a retrospective study of 236 elderly patients with early gastrointestinal cancer who were treated at the Department of Gastroenterology and Emergency of Lu'an Hospital Affiliated with Anhui Medical University between January 2022 and June 2025. Clinical data were systematically collected. Using stratified random sampling, patients were allocated to training and testing datasets in a 7:3 ratio. Feature selection was carried out via least absolute shrinkage and selection operator (LASSO) regression. Nine machine learning models were developed, and the model demonstrating the highest performance underwent SHAP (SHapley Additive exPlanations) analysis to enhance interpretability.

**Results:**

Among the 236 patients included in the analysis, 35 met the criteria for delayed post-ESD bleeding, resulting in an incidence of 14.8%. The remaining 201 patients did not experience delayed bleeding. LASSO regression identified seven variables associated with bleeding risk: large submucosal vessels, submucosal fibrosis, lesion diameter, lesion depth, procedure duration, use of anticoagulant medication, and hemoglobin level. Using these features, we constructed nine machine-learning models: K-nearest neighbors, support vector machine, extreme gradient boosting, approximate nearest neighbors, decision tree, light gradient boosting machine, random forest, extra trees, and gradient boosting machine. On the test set, the extra trees model performed best, with a sensitivity of 0.182, specificity of 0.983, F1-score of 0.286, and an area under the receiver-operating-characteristic curve of 0.879. SHAP analysis indicated that the features contributing most to the model's predictions were, in descending order: large submucosal vessels, lesion diameter, anticoagulant use, submucosal fibrosis, lesion depth, procedure duration, and hemoglobin level.

**Conclusion:**

We developed an interpretable Extra Trees model that effectively predicts the risk of post-ESD bleeding in elderly patients with early gastrointestinal cancer. This tool may aid clinicians in early identification of high-risk patients, thereby supporting targeted perioperative management and potentially improving clinical outcomes.

## Introduction

Gastrointestinal tumors are among the most serious diseases threatening human health worldwide. Epidemiological data show that gastrointestinal tumors account for over 25% of all newly diagnosed malignancies, and their associated mortality exceeds 30% (1). Due to the high incidence and poor prognosis of these diseases, early screening, precise diagnosis, and timely intervention have become crucial steps in improving patient survival outcomes.

Endoscopic submucosal dissection (ESD) has gained widespread acceptance and clinical application in the treatment of early gastrointestinal tumors due to its significant advantages, including complete lesion removal, minimal invasiveness, rapid postoperative recovery, and low complication rates (2–4). However, ESD requires high technical skill from the operator and involves relatively large resection areas, making delayed bleeding during wound healing a common and major postoperative complication. Reported incidence rates of delayed bleeding after ESD range from 1.8% to 15.6%, with higher risks observed in elderly patients, those with multiple comorbidities, and individuals on long-term anticoagulant therapy (5–7). Patients typically present with hematemesis or melena, and severe cases may lead to hemorrhagic shock or even life-threatening conditions. Therefore, accurate assessment of the risk for delayed bleeding before surgery or during the early postoperative period is clinically important for developing individualized monitoring and intervention strategies, thereby improving surgical safety and long-term outcomes.

Most existing studies on predicting post-ESD bleeding risk are based on traditional statistical models, which have limited variable dimensions and insufficient capacity to analyze nonlinear interactions among complex factors. In recent years, machine learning (ML) techniques, with their strong data fitting and pattern recognition capabilities, have shown broad application potential in areas such as disease prognosis prediction and complication risk assessment. By integrating multidimensional clinical data, laboratory indicators, and endoscopic features, ML can build highly predictive risk models. Furthermore, the introduction of model interpretability tools like SHapley Additive exPlanations (SHAP) enables intuitive analysis of black-box model outputs, helping to reveal the direction and magnitude of contributions from individual predictors, thereby enhancing the model's clinical acceptability and utility for decision-making (8–10).

Based on this, this study retrospectively collected clinical and endoscopic data from elderly patients with early gastrointestinal tumors in our hospital, constructed multiple machine learning prediction models to predict delayed bleeding after ESD, and conducted interpretability analysis on the model outputs using the SHAP method. The aim is to explore effective risk stratification tools, provide theoretical basis for early clinical warning, precise intervention, and individualized postoperative management, and thereby improve the efficacy of ESD surgery.

## Materials and methods

### Data source

In this retrospective observational study, we included 236 elderly patients with early gastrointestinal cancer who were admitted to the Department of Gastroenterology and Emergency, Lu'an Hospital affiliated with Anhui Medical University between January 2022 and June 2025. The cohort consisted of 77 cases of esophageal cancer, 159 cases of gastric cancer. There were 128 males and 108 females, with a mean age of 69.54 ± 5.75 years (range: 60–76).

Inclusion criteria were as follows: (1) Endoscopic histopathology confirmed early gastrointestinal cancer, with lesions confined to the mucosal or submucosal layer and without evidence of lymph node or distant metastasis (11, 12); (2) Age ≥60 years; (3) All patients underwent endoscopic submucosal dissection (ESD). Exclusion criteria included: (1) Contraindications to surgery; (2) History of previous gastrointestinal surgery; (3) Conversion to open surgery; (4) Coexistence of malignancy at other sites.

The study protocol was approved by the Institutional Ethics Committee of the hospital (2024LLKS-KY-047).

## Methods

### Treatment procedure

All patients underwent comprehensive preoperative assessment to confirm eligibility for endoscopic submucosal dissection (ESD). Procedures were performed by experienced endoscopists. Under appropriate anesthesia, patients were positioned suitably for the procedure. A standard endoscope was introduced, and the lesion was assessed for location, extent, and depth. Using a Dual knife, electrocautery markings were placed 3–5 mm beyond the lesion margin. A submucosal lifting solution—composed of normal saline, indigo carmine, sodium hyaluronate, and epinephrine—was injected to elevate the mucosal layer. A circumferential mucosal incision was then made approximately 0.5 cm outside the lesion, followed by sequential submucosal dissection in a layer-by-layer manner. Dissection was advanced bidirectionally until complete en-bloc resection was achieved. Intra- and post-procedural hemostasis was performed using coagulation-capable hemostatic forceps; visible vessels were treated as necessary. The resected specimen was retrieved for histopathological examination. After completion, the endoscope was withdrawn. Following the procedure, patients were maintained on a fasting regimen for 2–3 days and received standard proton-pump inhibitor therapy along with mucosal-protective agents.

### Definition of post-ESD bleeding

Delayed post-ESD bleeding was defined according to the following criteria occurring within 28 days after the procedure: (1) Clinically evident bleeding (melena, hematochezia, or hematemesis) accompanied by changes in vital signs (e.g., hypotension or tachycardia); (2) A decrease in hemoglobin level ≥20g/L from preoperative baseline; (3) Endoscopic confirmation of active bleeding or stigmata of recent hemorrhage; (4) Requirement for blood transfusion or repeat endoscopic or surgical hemostatic intervention (13, 14). All patients were monitored for 28 days following ESD. The occurrence of any of the above criteria was classified as a post-procedural bleeding event.

### Data collection

Based on the patients' relevant clinical records, the following data were collected:

(1) Baseline characteristics: sex, age, presence of diabetes, hypertension, family history of cancer, and use of anticoagulants (Anticoagulant, yes/no);
(2) Laboratory parameters: albumin (ALb), hemoglobin (Hb), platelet count (PLT), and carcinoembryonic antigen (CEA);
(3) Operative information: operation time (Operation time, < 60 min/≥60 min), intraoperative bleeding (yes/no), and wound management method (hemostatic clip applied/not applied);
(4) Endoscopic features: presence of submucosal thick vessels (Feature 1, yes/no), submucosal fibrosis (Feature 2, yes/no), lesion diameter (Feature 3, < 2 cm/≥2 cm), lesion depth (Feature 4, mucosal/submucosal), number of lesions (Feature 5, single/multiple), lesion location (Feature 6, esophagus/stomach), presence of ulceration (Feature 7, yes/no), and tumor morphology (Feature 8, flat or depressed/elevated).

### Model construction and evaluation

The patients were randomly divided into training and test sets in a 7:3 ratio using stratified random sampling. Feature selection was performed using the Least Absolute Shrinkage and Selection Operator (LASSO) (15). Based on the selected features, nine machine learning models were developed: K-Nearest Neighbors (KNN), Support Vector Machine (SVM), Extreme Gradient Boosting (XGBoost), Approximate Nearest Neighbors (ANN), Decision Tree (DT), Light Gradient Boosting Machine (LightGBM), Random Forest (RF), Extremely Randomized Trees (ET), and Gradient Boosting Machine (GBM).

Interpretability of the predictive models was achieved using the SHAP (SHapley Additive exPlanations) method, a unified approach that quantifies the contribution and impact of each feature on the final prediction. SHAP values explicitly indicate the magnitude and direction (positive or negative) of each predictor's influence on the outcome variable. Moreover, for each individual observation in the dataset, its prediction can be interpreted specifically based on the corresponding set of SHAP values. A nomogram was constructed based on the selected variables to facilitate clinical application, to evaluate the calibration of the prediction model, calibration curves were plotted, supplemented by the Hosmer-Lemeshow test. Decision curve analysis (DCA) was performed to quantify the net benefit across different threshold probabilities, thereby assessing the clinical utility of the prediction model (16, 17).

### Statistical analysis

Continuous data with normal distribution were expressed as mean ± standard deviation ($\bar{x}\;\pm$ *s*) and compared between groups using the independent samples *t*-test. Non-normally distributed continuous data were expressed as median (interquartile range) [*M* (P25, P75)] and compared using the Mann-Whitney *U* test. Categorical data were presented as frequency (percentage) and compared using the chi-square test. The models were evaluated using the receiver operating characteristic (ROC) curve, area under the curve (AUC), sensitivity, specificity, confusion matrix, positive predictive value (PPV), negative predictive value (NPV), and F1-score. The SHAP interpretability analysis of the machine learning models was performed and visualized using Python. A *P*-value < 0.05 was considered statistically significant.

## Results

### Baseline comparison

**Figure 1:**
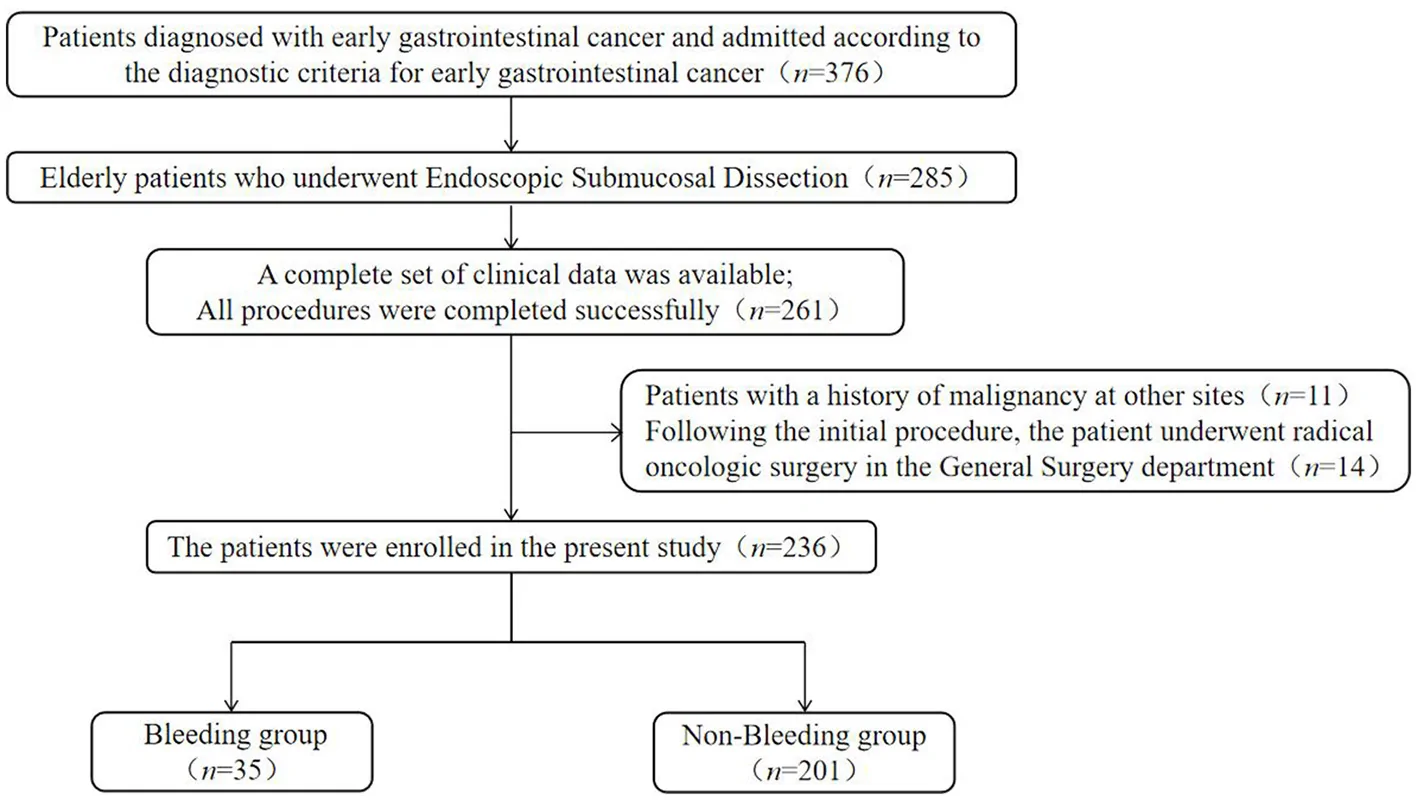
Flowchart of patient selection.

According to the inclusion and exclusion criteria, a total of 236 patients were enrolled in this study (see Figure 1 for the patient selection flowchart).Based on the post-ESD bleeding criteria, among the 236 patients, 35 experienced delayed bleeding and 201 did not, resulting in a bleeding incidence of 14.83% (35/236). Comparison of baseline characteristics between the bleeding and non-bleeding groups revealed significant differences (*P* < 0.05) in the presence of submucosal thick vessels, submucosal fibrosis, lesion diameter, lesion depth, number of lesions, hemoglobin level, use of anticoagulants, presence of ulceration, and wound management method. No statistically significant differences were observed for the remaining variables between the two groups (*P* > 0.05), as shown in Table 1.

**Table 1:** Comparison of baseline characteristics between the two groups ($\bar{x}\;$± *s, n*).

Variable	Total (*n* = 236)	Non-bleeding group (*n* = 201)	Bleeding group (*n* = 35)	Statistic	*p*-value
Sex, *n* (%)				0.550	0.458
Male	128 (54)	107 (53)	21 (60)		
Female	108 (46)	94 (47)	14 (40)		
Age (years)	69.54 ± 5.75	69.60 ± 5.74	69.20 ± 5.86	0.379	0.705
Hypertension, *n* (%)				1.068	0.301
No	104 (44)	96 (48)	8 (23)		
Yes	132 (56)	105 (52)	27 (77)		
Diabetes, *n* (%)				0.320	0.571
No	145 (61)	125 (62)	20 (57)		
Yes	91 (39)	76 (38)	15 (43)		
Operation time (min,), *n* (%)				2.773	0.096
< 60	125 (53)	111 (55)	14 (40)		
≥60	111 (47)	90 (45)	21 (60)		
Submucosal thick vessels, *n* (%)				27.251	0.000
No	163 (69)	152 (76)	11 (31)		
Yes	73 (31)	49 (24)	24 (69)		
Submucosal fibrosis, *n* (%)				15.370	0.000
No	182 (77)	164 (82)	18 (51)		
Yes	54 (23)	37 (18)	17 (49)		
Lesion diameter (cm), *n* (%)				13.576	0.000
< 2	115 (49)	108 (54)	7 (20)		
≥2	121 (51)	93 (46)	28 (80)		
Lesion depth, *n* (%)				13.063	0.000
Mucosal	214 (91)	188 (94)	26 (74)		
Submucosal	22 (9)	13 (6)	9 (26)		
Number of lesions, *n* (%)				16.111	0.000
single	205 (87)	182 (91)	23 (66)		
multiple	31 (13)	19 (9)	12 (34)		
Lesion location, *n* (%)				0.027	0.870
Esophagus	77 (33)	66 (33)	11 (31)		
Stomach	159 (67)	135 (67)	24 (69)		
Alb (g/L)	33.75 ± 3.33	33.75 ± 3.33	33.74 ± 3.35	0.016	0.987
Hb (g/L)	95.57 ± 12.08	96.25 ± 12.06	91.66 ± 11.60	2.089	0.038
CEA (ng/ml)	4.25 ± 1.14	4.21 ± 1.12	4.26 ± 1.15	0.968	0.663
PLT ( × 10?/L)	238 (184, 274)	237 (184, 274)	241 (190, 275.5)	0.821	0.803
Intraoperative bleeding, *n* (%)				0.051	0.821
No	159 (67)	136 (68)	23 (66)		
Yes	77 (33)	65 (32)	12 (34)		
Anticoagulant, *n* (%)				13.701	0.000
No	141 (60)	130 (65)	11 (31)		
Yes	95 (40)	71 (35)	24 (69)		
Ulceration, *n* (%)				6.203	0.013
No	175 (74)	155 (77)	20 (57)		
Yes	61 (26)	46 (23)	15 (43)		
Morphology, *n* (%)				1.176	0.278
Flat/depressed	191 (81)	165 (82)	26 (74)		
Elevated	45 (19)	36 (18)	9 (26)		
Family history, *n* (%)				0.638	0.425
No	181 (77)	156 (78)	25 (71)		
Yes	55 (23)	45 (22)	10 (29)		
Hemostatic clip, *n* (%)				6.873	0.009
Not applied	29 (12)	20 (10)	9 (26)		
Applied	207 (88)	181 (90)	26 (74)		

### Variable selection

**Figure 2:**
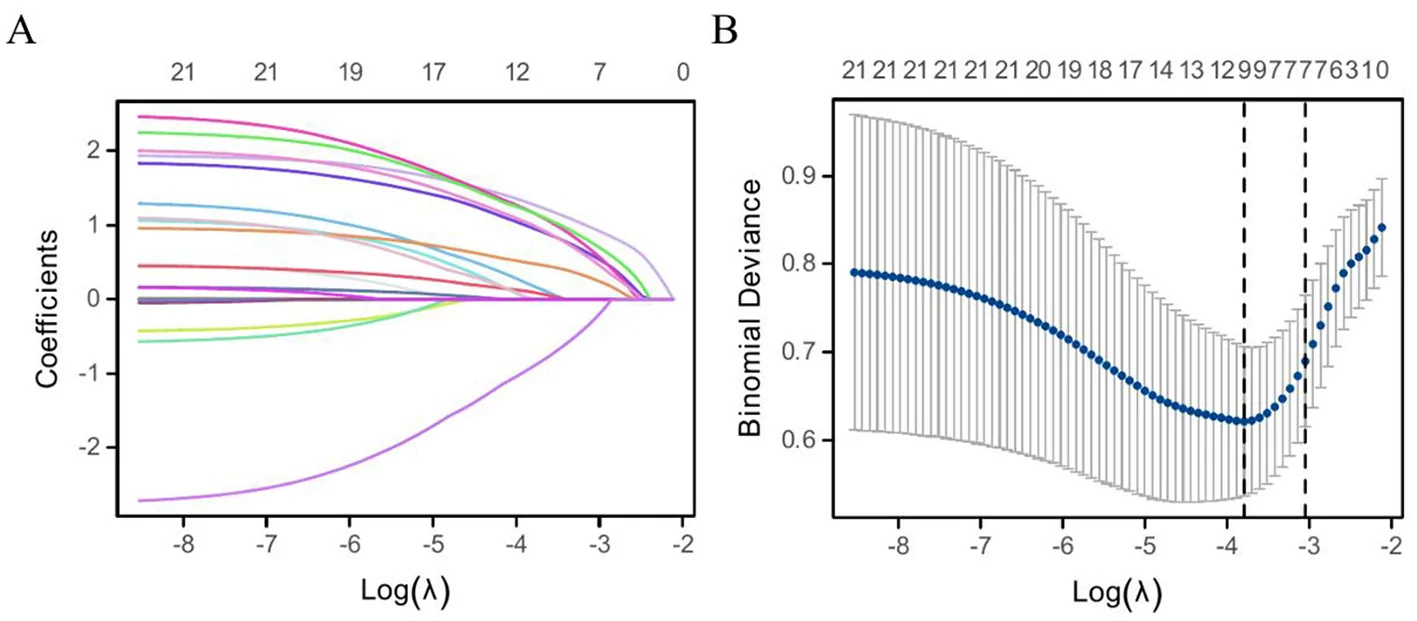
**(A)** Coefficient paths of variables selected by LASSO regression; **(B)** Parameter selection for the LASSO model via five-fold cross-validation and the minimum criterion, showing the binomial deviance plotted against log (λ).

**Figure 3:**
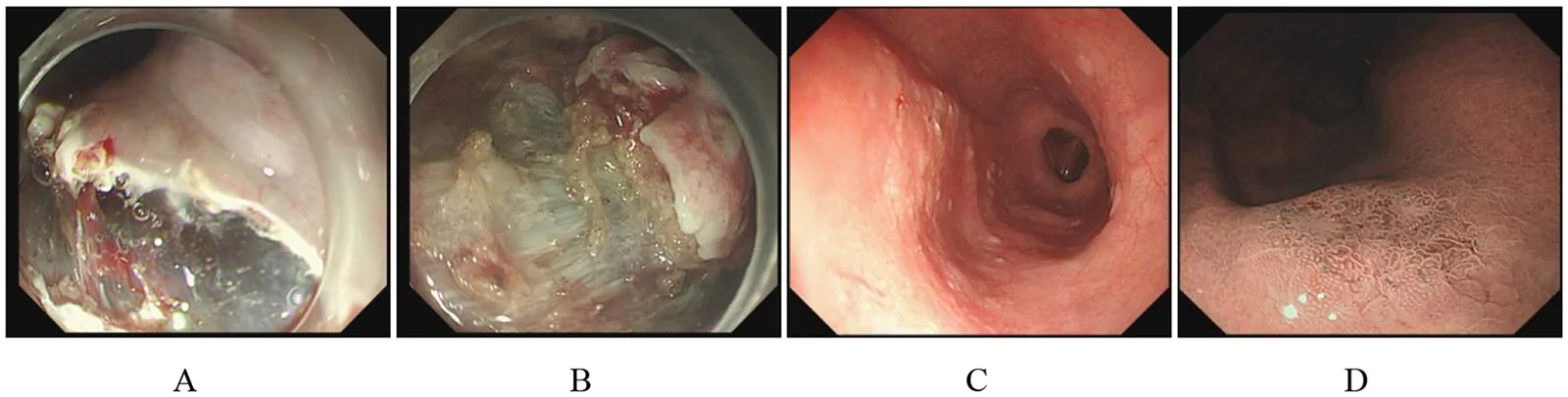
Four endoscopic features. **(A)** Submucosal thick vessels; **(B)** Submucosal fibrosis; **(C)** Lesion diameter ≥2 cm; **(D)** Lesion depth involving the submucosal layer.

LASSO regression was applied to reduce the dimensionality of the 21 candidate variables included in this study. Figure 2A illustrates the coefficient paths as a function of λ, and Figure 2B presents the feature selection profile using ten-fold cross-validation. The blue dots represent the mean cross-validated error for each model at a given λ, with the dashed line indicating the log (λ) value at the optimal λ. Using this optimal λ, seven variables were selected, including four endoscopic features: submucosal thick vessels (Feature 1), submucosal fibrosis (Feature 2), lesion diameter (Feature 3), and lesion depth (Feature 4). The remaining selected variables were operation time, use of anticoagulants (Anticoagulant), and hemoglobin (Hb) level. The four endoscopic features—submucosal thick vessels (Feature 1), submucosal fibrosis (Feature 2), lesion diameter (Feature 3), and lesion depth (Feature 4)—are visually presented in Figure 3.

### Machine learning model construction and evaluation

**Figure 4:**
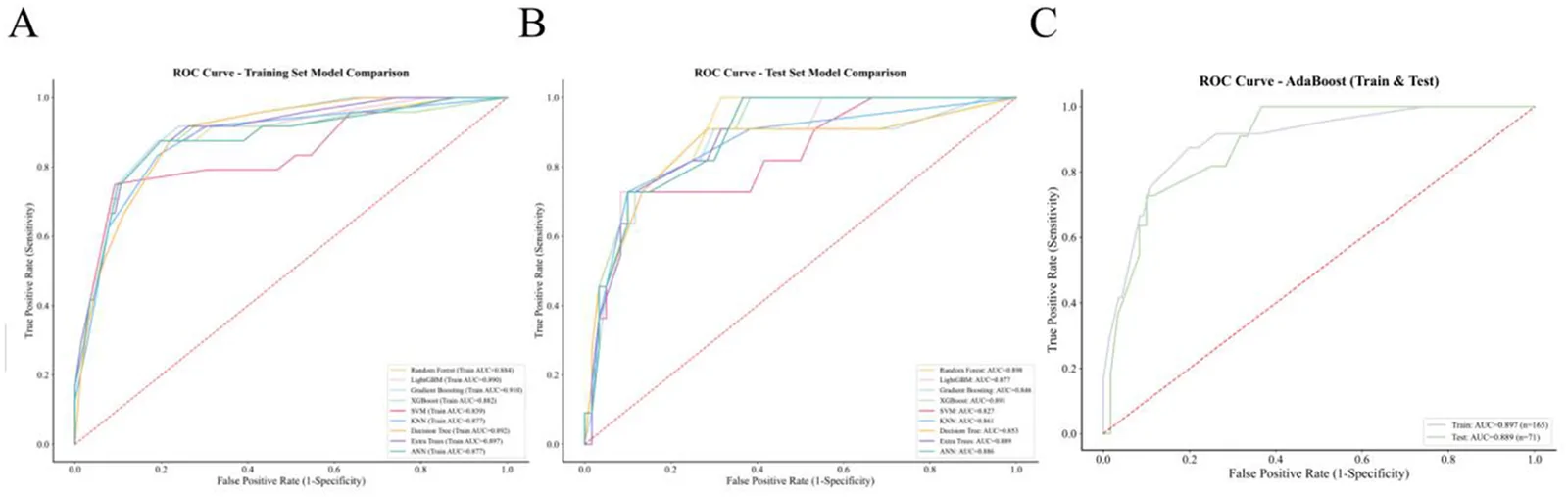
**(A)** ROC curves of the nine machine learning algorithms for prediction in the training set. **(B)** ROC curves of the nine machine learning algorithms for prediction in the test set. **(C)** ROC curves of the ET algorithm for prediction in both the training and test sets.

Based on the seven selected variables, nine machine learning algorithms were developed on both the training and test sets (Table 2). Figures 4A, B show the ROC curves of the nine models for predicting post-ESD bleeding in the training and test sets, respectively. The ET algorithm demonstrated the following performance: in the training set, sensitivity 0.292, specificity 0.986, F1-score 0.424, and ROC-AUC 0.885; in the test set, sensitivity 0.182, specificity 0.983, F1-score 0.286, and ROC-AUC 0.879. After comprehensively comparing the performance metrics of all machine learning models, the ET model was ultimately selected as the optimal model for predicting post-ESD bleeding in patients. The ROC curves of the ET algorithm in the training and test sets are presented in Figure 4C.

**Table 2:** Evaluation of prediction performance of the machine learning models.

Model	AUC	Sensitivity	Specificity	PPV	NPV	F1-score
Training set						
ANN	0.879	0.292	0.979	0.700	0.292	0.412
DT	0.879	0.333	0.972	0.667	0.333	0.444
KNN	0.873	0.125	1.000	1.000	0.125	0.222
LightGBM	0.879	0.292	0.979	0.700	0.292	0.412
RF	0.873	0.125	1.000	1.000	0.125	0.222
SVM	0.885	0.417	0.965	0.667	0.417	0.513
XGBoost	0.881	0.421	0.957	0.657	0.335	0.498
ET	0.885	0.292	0.986	0.778	0.292	0.424
498ptGBM	0.885	0.333	0.979	0.727	0.333	0.457
Test set						
ANN	0.873	0.364	0.967	0.667	0.364	0.471
DT	0.862	0.273	0.983	0.750	0.273	0.400
KNN	0.845	0.091	0.977	0.500	0.091	0.154
LightGBM	0.859	0.273	0.967	0.600	0.273	0.375
RF	0.845	0.091	0.983	0.500	0.091	0.154
SVM	0.873	0.364	0.957	0.667	0.375	0.469
XGBoost	0.863	0.364	0.967	0.671	0.364	0.471
ET	0.879	0.182	0.983	0.667	0.182	0.286
GBM	0.841	0.184	0.974	0.655	0.195	0.285

**Figure 5:**
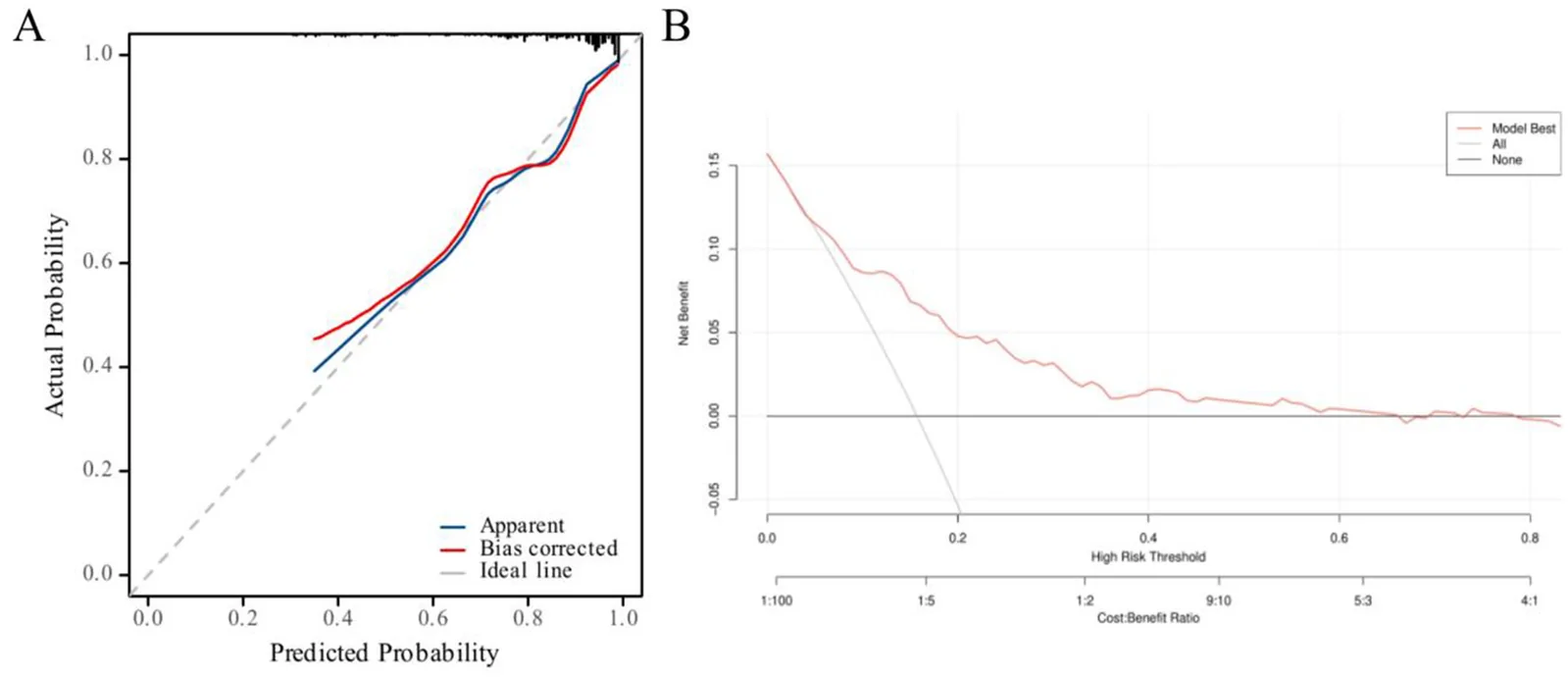
**(A)** The calibration plot of the ET model in the test set. **(B)** DCA of the ET model in the test set.

The calibration curve for the ET model in predicting bleeding probability in the test set demonstrated good agreement between predicted probabilities and observed outcomes. The Hosmer-Lemeshow test yielded a chi-square statistic of 8.206 (*P* = 0.414), indicating that the predicted probabilities were well calibrated with the actual incidence rates (Figure 5A). The decision curve analysis for the ET model in predicting bleeding probability in the test set is presented in Figure 5B. The decision curve demonstrated that when the threshold probability ranged from 6% to 65%, the net benefit derived from using the ET model for bleeding prediction was superior to both the “treat-all” and “treat-none” strategies.

### Interpretability analysis of the machine learning model

**Figure 6:**
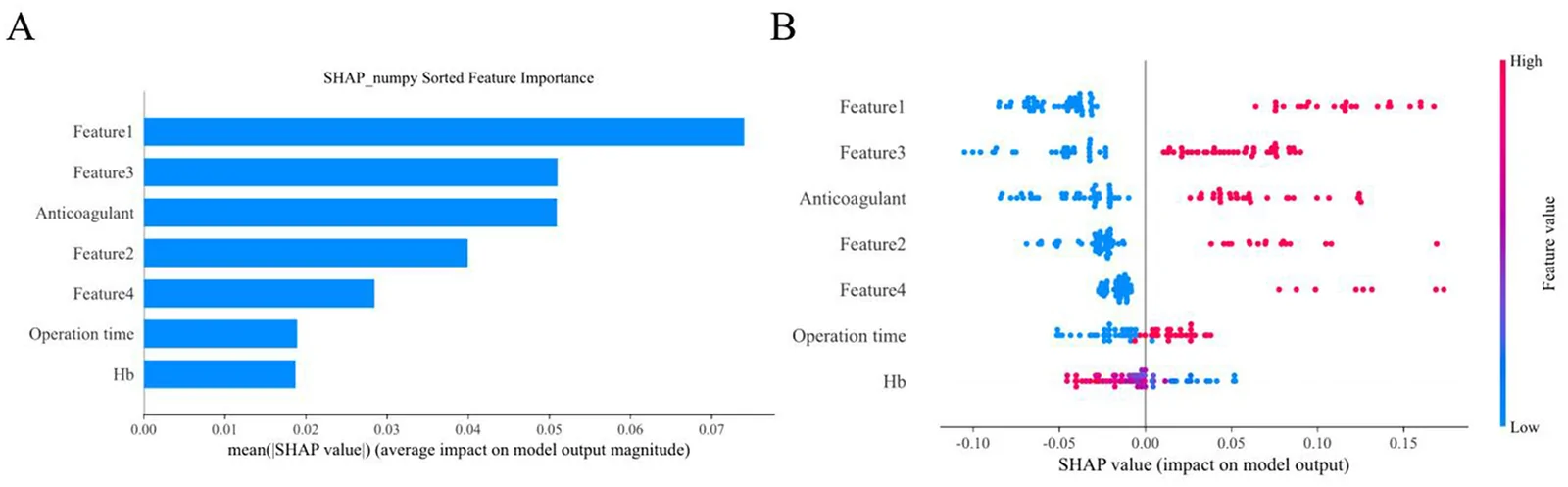
**(A)** SHAP feature importance bar plot; **(B)** SHAP feature importance beeswarm plot.

SHAP was used to interpret the optimal machine learning model (ET). As shown in the SHAP summary plots (Figures 6A, B), the contribution of each feature to the model was assessed by its mean absolute SHAP value and ranked in descending order. The seven variables were ranked by contribution as follows: submucosal thick vessels (Feature 1), lesion diameter (Feature 3), use of anticoagulants (Anticoagulant), submucosal fibrosis (Feature 2), lesion depth (Feature 4), operation time (Operation time), and hemoglobin (Hb) level.

**Figure 7:**
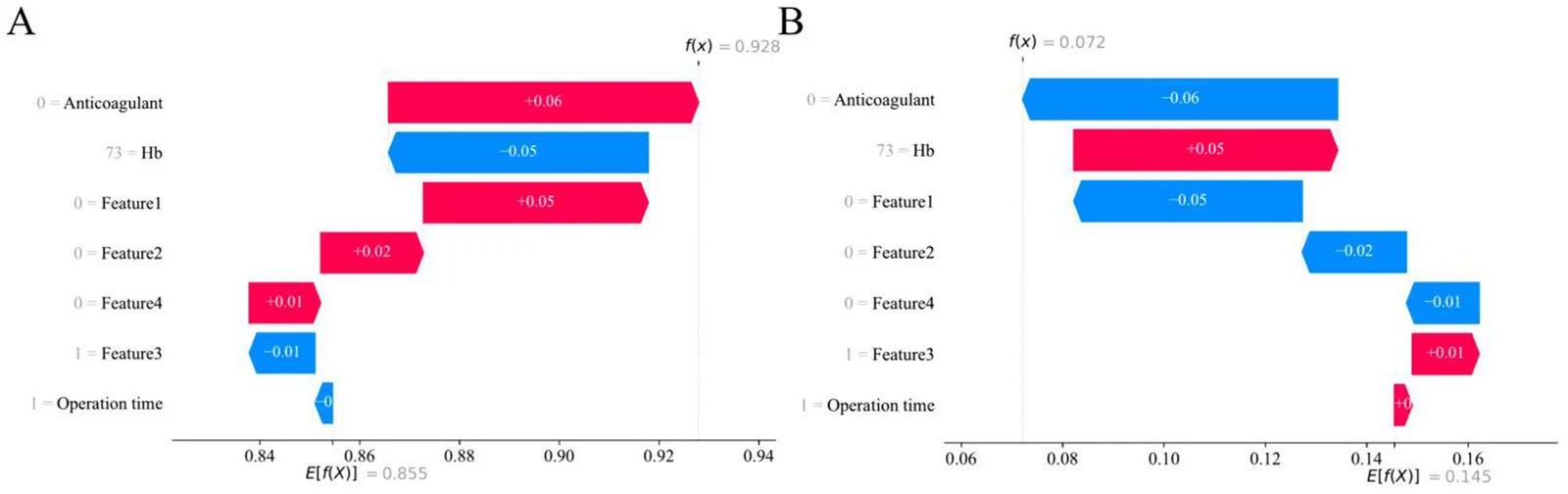
SHAP waterfall plots of two selected patients.

Figure 7 presents individual SHAP waterfall plots for **(A)** a patient with bleeding and **(B)** a patient without bleeding. The SHAP values illustrate the contribution of each feature to the prediction of post-ESD bleeding. Bold numbers indicate predicted probability [f_(x)_], the base value represents the model's default prediction without input, and f(x) corresponds to the log-odds ratio for each observation. Red features denote factors that increase the risk of post-ESD bleeding, while blue features represent factors that decrease the risk. The length of each arrow visually reflects the magnitude of the feature's influence (longer arrows indicate greater impact).

**Figure 8:**
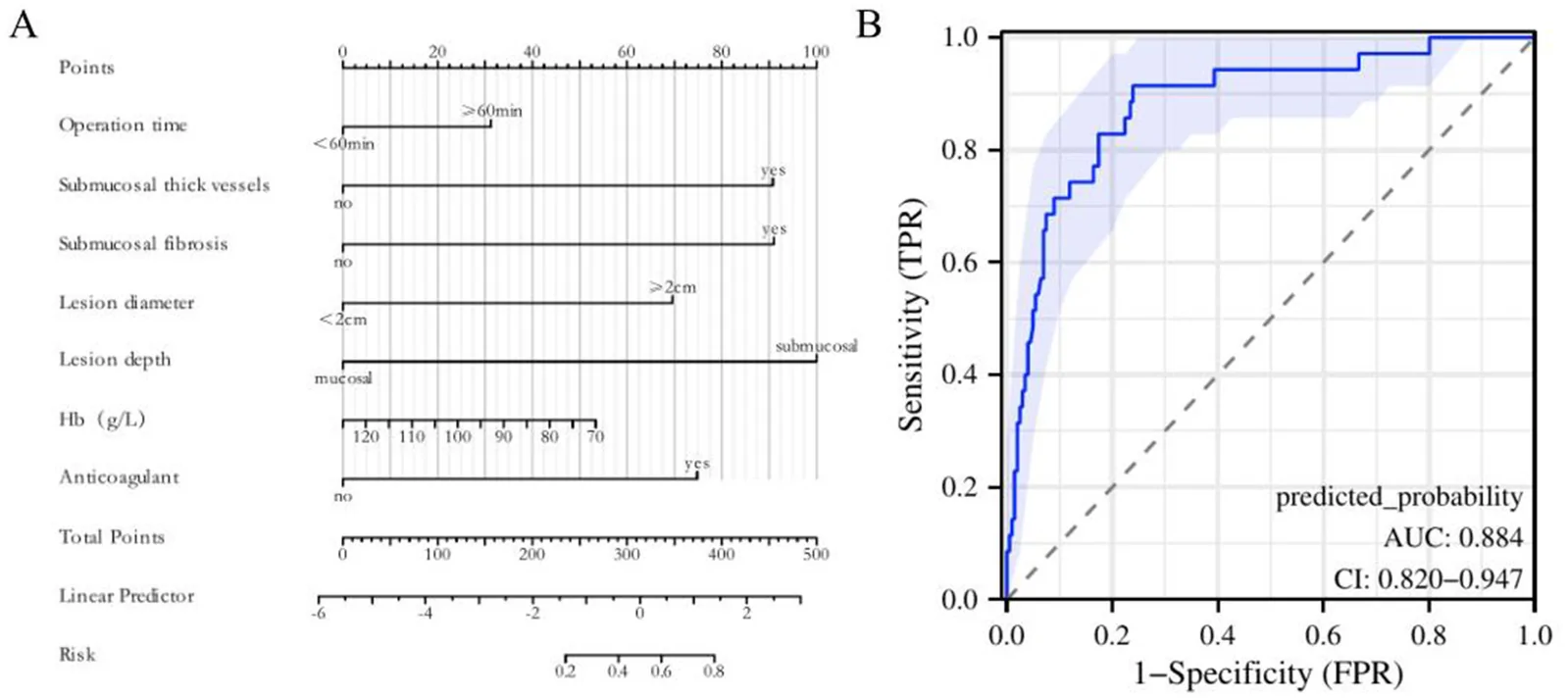
**(A)** Nomogram for predicting post-ESD bleeding risk based on seven selected features; **(B)** Nomogram-predicted ROC curve.

A nomogram was constructed based on the seven selected features (Figure 8A) to facilitate rapid estimation of a patient's risk of post-ESD bleeding. The area under the ROC curve predicted by the nomogram was 0.884 (95% CI: 0.820–0.947).

**Figure 9:**
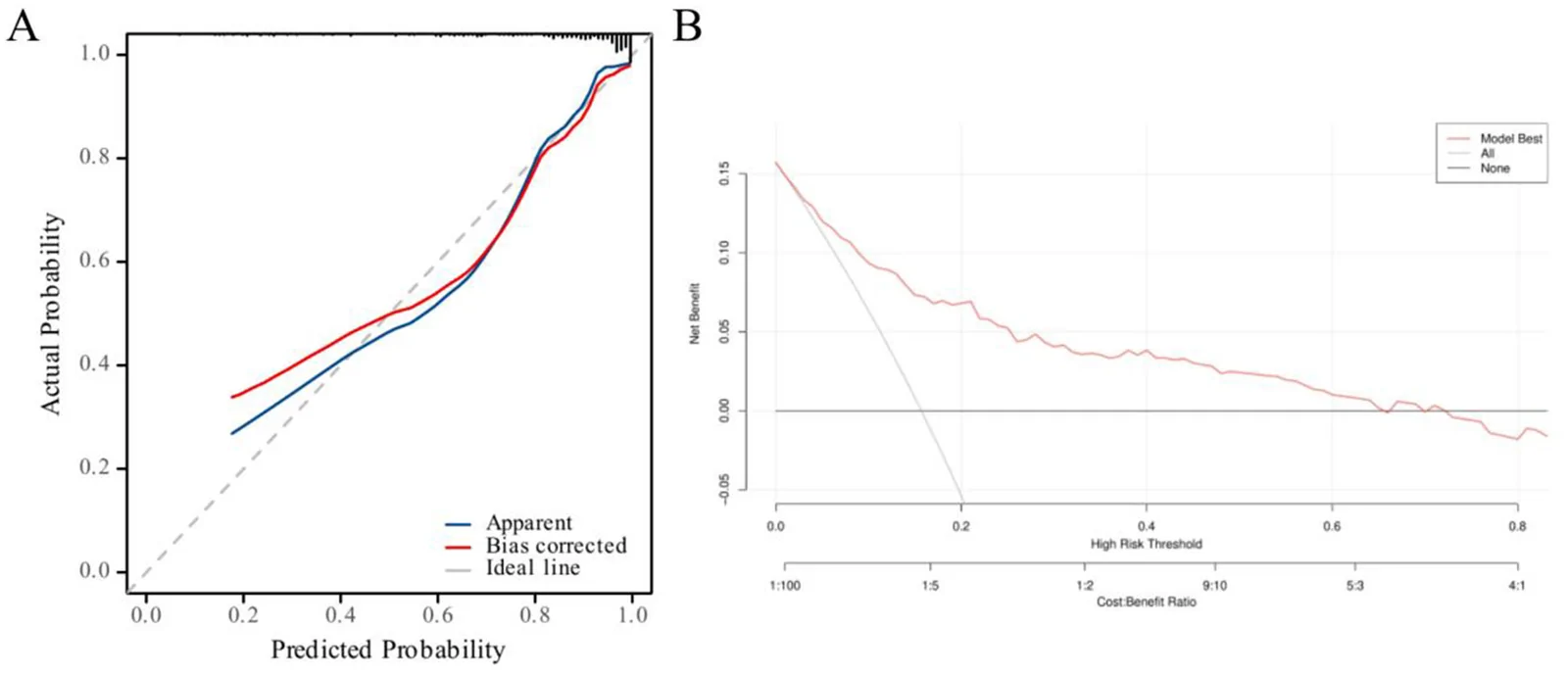
**(A)** The calibration plot of the nomogram model in the dataset. **(B)** DCA of the nomogram model in the dataset.

The calibration curve of the nomogram model for predicting bleeding probability in the dataset demonstrated satisfactory agreement between predicted probabilities and observed outcomes. The Hosmer-Lemeshow test yielded a chi-square statistic of 4.891 (*P* = 0.769), indicating that the model's predicted probabilities were well calibrated with the actual incidence rates (Figure 9A). The decision curve analysis for the nomogram model in predicting bleeding probability in the dataset is presented in Figure 9B. The decision curve demonstrated that when the threshold probability ranged from 5% to 70%, the nomogram model yielded a higher net benefit for bleeding prediction compared with the “treat-all” and “treat-none” strategies.

## Discussion

Early gastrointestinal cancer is defined as a malignant epithelial tumor confined to the mucosal or submucosal layer of the gastrointestinal tract, regardless of regional lymph node involvement and without invasion into the muscularis propria. ESD is the current standard of care for such lesions, allowing curative en bloc resection of mucosal and submucosal tumors with lower invasiveness, faster recovery, and shorter hospitalization compared with conventional surgery (18, 19).

However, the technical complexity of ESD confers a substantial risk of postprocedural bleeding, which may adversely affect treatment outcomes (20). In the present study, according to the predefined criteria for delayed bleeding, bleeding events occurred in 35 of 236 elderly patients (14.83%), while 201 patients did not bleed. This finding indicates that elderly patients with early gastrointestinal cancer remain at considerable risk of bleeding after ESD (21). Early identification of patients at high bleeding risk is therefore essential to guide preventive strategies, reduce bleeding rates, and improve prognosis (22, 23). While prior studies have mainly focused on clinical risk factors, they often lack detailed endoscopic intraoperative characteristics. By contrast, this study developed and applied a machine learning model based on endoscopic features to predict delayed bleeding after ESD in elderly patients, which may help clinicians identify high-risk individuals early and facilitate timely intervention.

In this study, significant differences were observed between the bleeding and non-bleeding groups in terms of submucosal thick vessels, submucosal fibrosis, lesion diameter, lesion depth, number of lesions, hemoglobin level, anticoagulant use, ulceration, and wound management method. Using LASSO regression on 21 candidate variables, seven were selected: four endoscopic features (submucosal thick vessels, submucosal fibrosis, lesion diameter, and lesion depth) along with operation time, anticoagulant use, and hemoglobin level. Thick submucosal vessels, with their larger caliber and higher intraluminal pressure, increase the risk of post-procedural bleeding. Even if adequately coagulated during ESD, the resulting eschar is vulnerable to detachment from exposure to digestive fluids or mechanical friction after the procedure, which can lead to re-bleeding. Submucosal fibrosis obscures tissue planes and increases the likelihood of iatrogenic injury to deeper vessels during dissection. Additionally, reduced tissue elasticity compromises secure vessel closure, while impaired local perfusion and delayed wound healing promote the formation of fragile neovessels that are prone to rupture (24, 25). Larger lesions require more extensive dissection, exposing a greater number of submucosal vessels and raising the probability of transecting major or branching vasculature. Subsequent wound contraction may then dislodge the coagulated eschar, resulting in re-bleeding. Submucosal invasion indicates that the tumor has penetrated the muscularis mucosa, making deeper vascular networks more vulnerable during dissection. Moreover, tumor-related inflammatory reactions and peritumoral fibrosis can diminish the efficacy of electrocautery, further elevating the risk of post-ESD bleeding (26).

Prolonged procedure time exacerbates local tissue edema and inflammatory responses. The release of inflammatory mediators such as prostaglandins and nitric oxide during extended resection increases vascular permeability and inhibits platelet aggregation, thereby elevating the risk of postoperative bleeding. The use of anticoagulant or antiplatelet agents impairs normal hemostatic mechanisms (27). Whether through inhibition of platelet function (e.g., aspirin) or reduction of coagulation factor activity (e.g., warfarin), these medications compromise thrombus stability during and after the procedure (28). Even when intraoperative hemostasis appears adequate, the persistent pharmacologic effect may lead to vessel recanalization or ongoing oozing in the postoperative period (29). Lower hemoglobin levels frequently reflect chronic anemia or malnutrition. In such patients, diminished tissue oxygenation impairs mucosal repair and delays wound healing. Consequently, exposed submucosal vessels remain susceptible to digestive fluids and mechanical irritation for a prolonged duration, increasing the likelihood of delayed bleeding (30, 31). Using the seven variables selected by LASSO regression, nine machine learning models were constructed. After comprehensive evaluation of performance metrics, the Extremely Randomized Trees (ET) model demonstrated superior and consistent predictive accuracy in both training and test datasets, indicating strong discriminative ability and adequate calibration for identifying elderly patients at high risk of delayed bleeding after ESD for early gastrointestinal cancer.

Interpretability of the Extremely Randomized Trees (ET) model was analyzed using SHAP (SHapley Additive exPlanations). SHAP values quantify the contribution of individual features to model predictions and help visualize potential nonlinear relationships between predictors and outcomes. This approach enhances the transparency and trustworthiness of machine learning in medical contexts, offering clinicians intuitive and reliable decision support while addressing the “black-box” limitation often associated with clinical prediction models (32, 33). In the present analysis, SHAP values ranked the seven selected features by contribution in descending order as: submucosal thick vessels, lesion diameter, anticoagulant use, submucosal fibrosis, lesion depth, operation time, and hemoglobin level. A corresponding nomogram integrating these features was constructed to facilitate rapid clinical estimation of post-ESD bleeding risk. In summary, this study developed an efficient and interpretable machine-learning model for predicting delayed bleeding risk after ESD in elderly patients with early gastrointestinal cancer. Such a tool may assist clinicians in early identification of high-risk individuals, enabling timely preventive measures—such as meticulous dissection combined with clip or suture reinforcement, topical hemostatic agents, preoperative optimization of coagulation status, and intensified postoperative monitoring—thereby potentially improving procedural safety and clinical outcomes (34–36).

This study has several limitations. First, the data were derived from a single-center retrospective observational design without an external validation cohort, which may introduce selection bias and thereby limit the generalizability of our findings to broader patient populations and diverse clinical settings. Second, owing to the constrained sample size, we were unable to perform separate subgroup analyses for esophageal and gastric lesions. Given the well-recognized differences between these two anatomical sites in terms of mucosal architecture, vascular supply, and postoperative management protocols, the combined analysis may have obscured site-specific risk factors and compromised the predictive performance of the model. Consequently, the integrated analytical approach employed in this study should be considered exploratory in nature. Future investigations involving multicenter designs and larger sample sizes are warranted to validate and refine the current model, with particular emphasis on stratified analyses according to lesion location.

## Conclusions

In summary, the Extremely Randomized Trees model developed in this study demonstrated accurate predictive performance for delayed bleeding risk after endoscopic submucosal dissection in elderly patients with early gastrointestinal cancer. These findings offer a potential basis for early clinical risk stratification and tailored intervention to improve procedural outcomes. SHAP interpretability analysis identified the following order of feature importance: submucosal thick vessels, lesion diameter, anticoagulant use, submucosal fibrosis, lesion depth, operation time, and hemoglobin level.

## Data Availability

The raw data supporting the conclusions of this article will be made available by the authors, without undue reservation.
